# Efficient keystone species identification strategy based on tabu search

**DOI:** 10.1371/journal.pone.0285575

**Published:** 2023-05-11

**Authors:** Chuanjin Fan, Donghui Zhu, Tongtong Zhang, Ruijia Wu

**Affiliations:** 1 School of Mathematics and Statistics, Shandong University, Weihai, Shandong, China; 2 SDU-ANU Joint Science College, Shandong University, Weihai, Shandong, China; 3 School of Law, Weihai, Shandong University, Weihai, Shandong, China; Southwest Jiaotong University, CHINA

## Abstract

As species extinction accelerates globally and biodiversity declines dramatically, identifying keystone species becomes an effective way to conserve biodiversity. In traditional approaches, it is considered that the extinction of species with high centrality poses the greatest threat to secondary extinction. However, the indirect effect, which is equally important as the local and direct effects, is not included. Here, we propose an optimized disintegration strategy model for quantitative food webs and introduced tabu search, a metaheuristic optimization algorithm, to identify keystone species. Topological simulations are used to record secondary extinctions during species removal and secondary extinction areas, as well as to evaluate food web robustness. The effectiveness of the proposed strategy is also validated by comparing it with traditional methods. Results of our experiments demonstrate that our strategy can optimize the effect of food web disintegration and identify the species whose extinction is most destructive to the food web through global search. The algorithm provides an innovative and efficient way for further development of keystone species identification in the ecosystem.

## Introduction

Ecosystems have been severely damaged in recent years by substantial increases in population density, emissions of industrial carbon dioxide and toxic pollutants, as well as habitat destruction. As a result, a large number of species became extinct, and biodiversity has dramatically declined. Species extinction poses a threat to the global ecosystem. In complex ecological networks, the extinction of some species, i.e., primary extinction, might result in the extinction of others, i.e., secondary or cascading extinction, due to the interdependence of species [1]. For example, deforestation and frequent wildfires in the Amazon rainforest have led to the destruction of habitats and food chains, resulting in a significant loss of biodiversity [2]. Consequently, conserving biodiversity helps to reduce species extinctions and secondary extinctions while protect ecosystems at the same time. However, insufficient resources—financial, human and energy—make it challenging to monitor population changes of all species in time to protect them from existential crises.

Keystone species are species in ecosystems that play a critical role in maintaining biodiversity and its structure, function and stability. The loss of keystone species will have a higher influence on the function and structure of ecosystems than the loss of other species, and is likely to trigger more secondary extinctions [3–6]. Therefore, the conservation of keystone species plays an important role in saving numerous other species while contributing to the conservation of resources. Identifying keystone species helps to set priorities and conservation targets, and serves as a guide for species and biodiversity conservation [7, 8]. In ecosystems, biodiversity loss has a significant impact on food web structure, including secondary and cascading extinctions [1]. The structure of a food web can be described as a network, with species represented as nodes and predator-prey relationships between species constituting directed links. These directed links begin with the prey and end with the predator. Such network-based analysis is a crucial tool for identifying keystone species [7, 9, 10]. Centrality in network analysis identifies the most critical nodes in a network [11]. Ranking species according to centrality has become a generally acknowledged method for prioritizing species for conservation [1, 12–16]: species at the center play a mediating role in the interactions between those at the margins and should therefore be considered the most important species. Previous research has used betweenness centrality [17], eigenvector centrality [18], closeness centrality [19], and through-flow centrality [20] for refinement, providing simple but effective methods for identifying the importance level of species.

However, a huge drawback in most network studies is the wide use of binary (i.e., presence or absence based on energy interactions) food webs. Though binary food webs are convenient in terms of data collection, it is increasingly recognized that such qualitative network structures are often uninformative [21]. Nutritional interactions of different strengths may have qualitatively distinct effects on food web properties, so increasing numbers of research now incorporate weighted networks [20–22], which can drastically modify conclusions about the importance of nodes [7, 23]. Across many quantitative food webs, link weights (i.e., the strength of energy interactions) are estimated based on the number of individuals, biomass, or carbon flux between species or compartments [20–22, 24]. These quantitative methods are more reliable than topological methods. However, in weighted networks, the centrality-based methods above only calculate local and direct effects and are unable to detect indirect effects, which have been shown to be equally significant [25]. For example, the nodes with the greatest connections are not always the most destructive ones [18]. Therefore, researchers have concentrated on the nodal effects of the entire network to find a globally optimal method that better understands their indirect effects.

As a metaheuristic algorithm for solving global optimal problems, tabu search [26, 27] is based on a neighborhood search approach that uses different types of memory and neighborhood strategies to direct the iterative improvement of the search. Recent studies have applied tabu search for the traditional network disintegration problem and achieved desirable outcomes. For example, Deng and Wu [28] studied the optimal disintegration strategy for undirected binary networks on the basis of tabu search, and Yu Yang et al. [29] extended it to directed networks. Results of both studies demonstrate that the disintegration strategy in accordance with tabu search performs significantly better than the conventional disintegration strategy. The distinctive characteristic of tabu search is that it uses memory to steer local search to jump out of the local optimum. By introducing a variational operation, when confronted with a local optimum, the ultimate result will move to the vicinity of the best neighborhood solution, even if temporary deteriorations of the objective function value may occur during the process. To avoid loops, a tabu list is employed, which keeps track of the attributes of recent moves and prohibits their repeat.

Here, we present a food web disintegration strategy based on tabu search to determine the globally optimal attack strategy for quantitative food webs and thus evaluate the importance of species on food web robustness. In the current study, we investigated the robustness of 12 real food webs and compared the performance of tabu search-based algorithm with centrality and eigenvector-based algorithms in identifying keystone species. Experiments show that the keystone species identification strategy based on tabu search performs significantly better than other methods.

The remainder of this paper is organized as follows. First, we provide an optimization model for disintegration strategy in quantitative food webs, and investigate the method for solving the optimization model based on tabu search. Then, the data used in this study are described, along with the simulation method and measurement metrics for measuring species extinction. Subsequently, we evaluate the proposed strategy on several food webs and analyze the results. Finally, we conclude the work and discuss some possible extensions.

## Materials and methods

### Optimization model for disintegration strategy in quantitative food webs

According to the definition of keystone species, a species is more important in a food web if its removal causes more secondary extinctions. Therefore, our goal is to find out which species have the most significant impact on the whole food web after removal, and an optimization model could be developed. First, we make the following conventions.

1. Consider a quantitative food web *G* = (*V*, *E*), where *V* represents the set of species (nodes) in the food web, *v*_*i*_ represents the species, and *N* denotes the number of *V*. *E* denotes the set of links (edges) for energy flow in the food web, *e*_*ij*_ denotes the energy route flowing from species *i* to species *j*, and *w*_*ij*_ denotes the energy value flowing from species *i* to species *j*. The in-degree of node *i* implies the energy obtained by species *i*, and the out-degree of node *i* indicates the energy flowing out of species *i*.
2. All the edges connected with *v*_*i*_ will vanish simultaneously after breaking a node *v*_*i*_ in *V*.
3. The in-degree of node *i* is $\sum_{j=1}^{N}w_{ji}$. A node is eliminated if, after a species extinction, the in-degree of node *i* as a percentage of the in-degree of node *i* in the initial food web falls below extinction threshold *t*.
4. The value of *x*_*i*_ represents the state of node *v*_*i*_ in the food web, with 0 indicating destroyed and 1 indicating uncorrupted.
5. The species removal strategy is denoted by *X*_*ind*_ = [*x*_1_, *x*_2_, …, *x*_*N*_], where *x*_*i*_ = 0 or 1, *i* = 1, 2, …, *N*, *n* represents the number of species removed.

Based on the assumptions above, the optimization model for the disintegration strategy is obtained:

$$\begin{array}{cc} & \text{max}\;F(X_{ind}=[x_{1},x_{2},\ldots ,x_{N}])\\ \;\text{s.t.}\; & \left\{ \begin{array}{c} \sum_{i=1}^{N}x_{i}=N-n\\ x_{i}=0\;\text{or}\;1,i=1,2,\ldots ,N \end{array}\text{.}\;\right. \end{array}$$ (1)

For any species removal strategy *X*_*ind*_, we have $\sum_{i=1}^{N}x_{i}=N-n$, where *n* denotes the number of species removed. We use *G*_0_ to denote an unbroken food web and *G* to denote a food web where some species have been removed by a strategy *X*_*ind*_. Also we use *F* to signify the objective function and *f*(*G*) to signify the number of surviving species in the food web after a species extincts. Thus the degree of disintegration *F*(*X*_*ind*_) can be represented by *F*(*X*_*ind*_) = *f*(*G*_0_) − *f*(*G*). Based on the assumptions above, *f*(*G*) is calculated as:

$$\begin{array}{c} f\left(G\right)=\sum_{i=1}^{N}I\left(\frac{\sum_{j=1}^{N}w_{ji}^{{}^{\prime }}}{\sum_{j=1}^{N}w_{ji}}\right) \end{array}$$ (2)

where *I*(*x*) is a schematic function, *I*(*x*) = 0 when *x* is below the extinction threshold *t* and *I*(*x*) = 1 when *x* is greater than the threshold *t*. $\sum_{j=1}^{N}w_{ji}$ is the in-degree of *v*_*i*_ in the initial network, and $\sum_{j=1}^{N}w_{ji}^{{}^{\prime }}$ is the in-degree of *v*_*i*_ in the present network.

For any subgraph *G* of *G*_0_, since *N* < *N*_0_, *f*(*G*) < *f*(*G*_0_), *F* ≥ 0. The more species become extinct, the lower the ratio of the in-degree of node *v*_*i*_ in *G* to that in *G*_0_, the smaller *f*(*G*), the larger *F*(*X*_*ind*_), and the greater the disintegration is.

### Removal approach based on tabu search

It can be inferred from the objective function that the model above is a nonlinear optimization model. If the solution is performed with a traversal search, the computation is $C_{N}^{n}=N!/\left(n!\left(N-n\right)!\right)$, which increases sharply as *N* and *n* increase. Therefore, the traversal search approach is almost impossible for large food webs, so we use a metaheuristic algorithm, tabu search, to solve the problem.

Tabu search is a metaheuristic search algorithm, which is an effective tool for solving global optimization problems [27]. It uses a local search method iteratively search for the optimal solution among the neighborhood of the current solution as the next solution until no better solution can be found. By incorporating mutation operations, it enhances the ability to evade the local optimum and enhances the ability to find the global optimum. Tabu search is comprised of six components that are essential for its operation: objective function, movement mechanism, prohibition rule, tabu list, expectation criterion, and termination criterion. The objective function is a measure used to evaluate neighbors and judge their merits. In the present study, the food web disintegration degree *F*(*X*_*ind*_) is employed as the objective function. The move mechanism reflects the search for the best solution. The fundamental principle of the move mechanism is first to generate an initial solution, then iteratively search its neighborhood and select the optimal solution for the next step. To avoid search loops and local optimums, a tabu list (*T*_*list*_) recording forbidden solutions is employed. The most critical factor in its design is the tabu step length (*L*, the number of iterations after which the object’s tabu expires). Meanwhile, the aspiration criterion is a moderate relaxation of the tabu table for a given length, where old tabu objects are gradually exited as new tabu objects enter and can be revisited. A move should accept when its result is better than the optimal neighborhood solution obtained by unforbidden moves and the optimal history solution achieved without the restriction of the tabu table. The termination criterion is a rule of stopping, which is set according to the upper limit of the number of iterations (*T*_*max*_).

**Fig 1:**
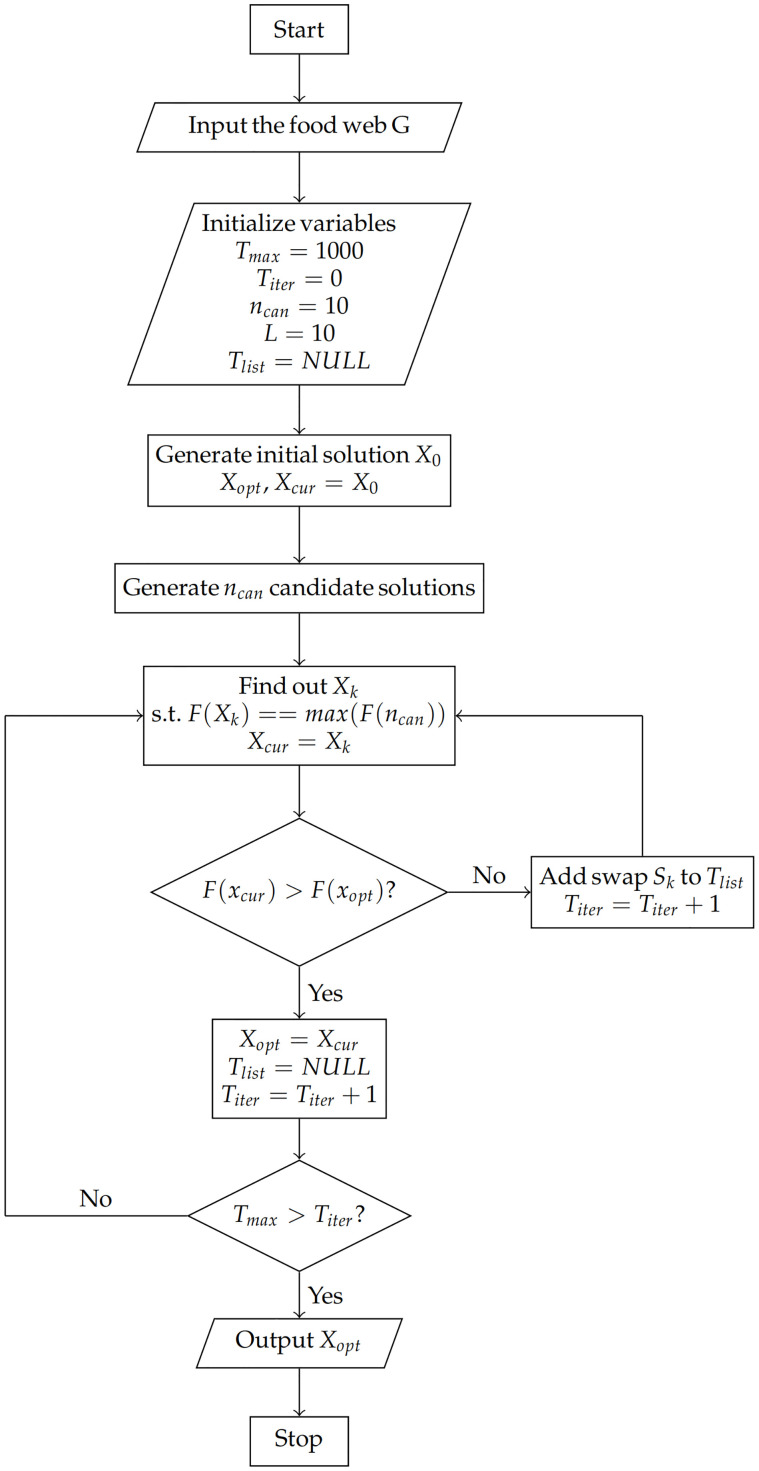
Flow chart of tabu search.

The flowchart for solving the best disintegration strategy of food webs based on tabu search is shown in Fig 1, with the following detailed steps:

- Step 1. First, enter the food web data into the program. Add a virtual node *v*_1_ to represent the external environment, and add edges from *v*_1_ to *v*_2_, …, *v*_*N*+ 1_ as well as the edges from *v*_2_, …, *v*_*N*+1_ pointing to *v*_1_, indicating the energy that *v*_2_, …, *v*_*N*+1_ receives and flows into the external environment.
- Step 2. Initialize the maximum number of iterations(*T*_*max*_), the maximum number of candidate solutions (*n*_*can*_), the length of the tabu list (*L*), the tabu list (*T*_*list*_ = *NULL*), and current iteration (*T*_*iter*_ = 0).
- Step 3. Randomly generate an initial solution *X*_0_ = [*x*_1_, *x*_2_, …, *x*_*N*_, *x*_*N*+1_]. The specific operation is to randomly select n different nodes in *v*_2_, …, *v*_*N*+1_ and let their corresponding x values be 0. The historical optimal solution and the current solution are noted as *X*_*opt*_ and *X*_*cur*_, respectively. Initialize *X*_*opt*_, *X*_*cur*_ = *X*_0_.
- Step 4. Initialize swap list (*S*_*list*_ = *NULL*). Generate *n*_*can*_ candidate solutions *X*_1_, …, *X*_*ncan*_ based on *X*_*cur*_. The specific operation is to randomly select a node *y*_0_ with value 0 in *X*_*cur*_, and then randomly select a node *y*_1_ (*y*_1_ ≠ *v*_1_) with value 1 among the remaining nodes with. Denote the set of *y*_0_ and *y*_1_ ({*y*_0_, *y*_1_}) as a swap *S*_*i*_. If *S*_*i*_ is in *S*_*list*_ or *T*_*list*_, this generated solution is invalidated and retry to generate a solution. If not, *S*_*i*_ is added into *S*_*list*_, and the candidate solution is generated successfully. Repeat the operation above until the number of generated solutions is *n*_*can*_, then *S*_*list*_ = *NULL*.
- Step 5. Calculate *F*(*X*_*ind*_), *ind* = 1, …, *n*_*can*_ separately, and the *X*_*ind*_ corresponding to the maximum value of *F*(*X*_*ind*_) is noted as *X*_*k*_. Then *X*_*cur*_ = *X*_*k*_, and record the swap *S*_*k*_ when generating solution *X*_*k*_.
- Step 6. If *F*(*X*_*cur*_) > *F*(*X*_*opt*_), then *T*_*list*_ = *NULL* and *X*_*opt*_ = *X*_*cur*_. Otherwise, add *S*_*k*_ to *T*_*list*_ and delete the earliest added swap if *T*_*list*_ is full. In either case, *T*_*iter*_ = *T*_*iter*_ + 1.
- Step 7. If *T*_*iter*_ > *T*_*max*_, terminate the iteration and output *X*_*opt*_. Otherwise, go back to Step 4, and repeat the operation above until *T*_*iter*_ > *T*_*max*_.

**Fig 2:**
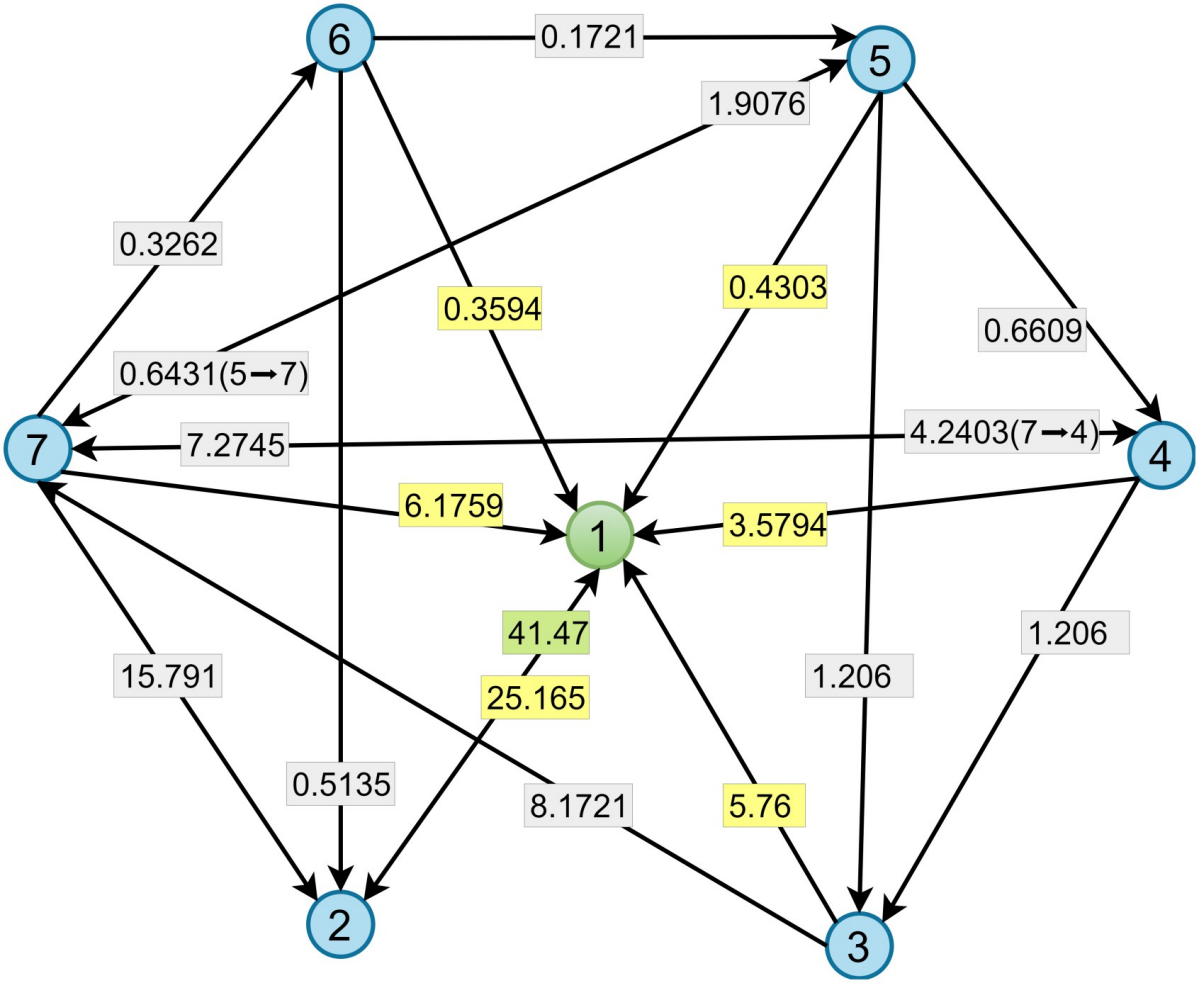
A quantitative food web example with 6 species and 19 weighted links. Virtual node *v*_1_ represents the external environment. The link from *v*_1_ to *v*_2_ with a green weight represents the energy flow that the food web receives from the external environment, and the links from *v*_2_, …, *v*_7_ pointing to *v*_1_ with yellow weights indicate energy flows from the food web into the external environment.

**Fig 3:**
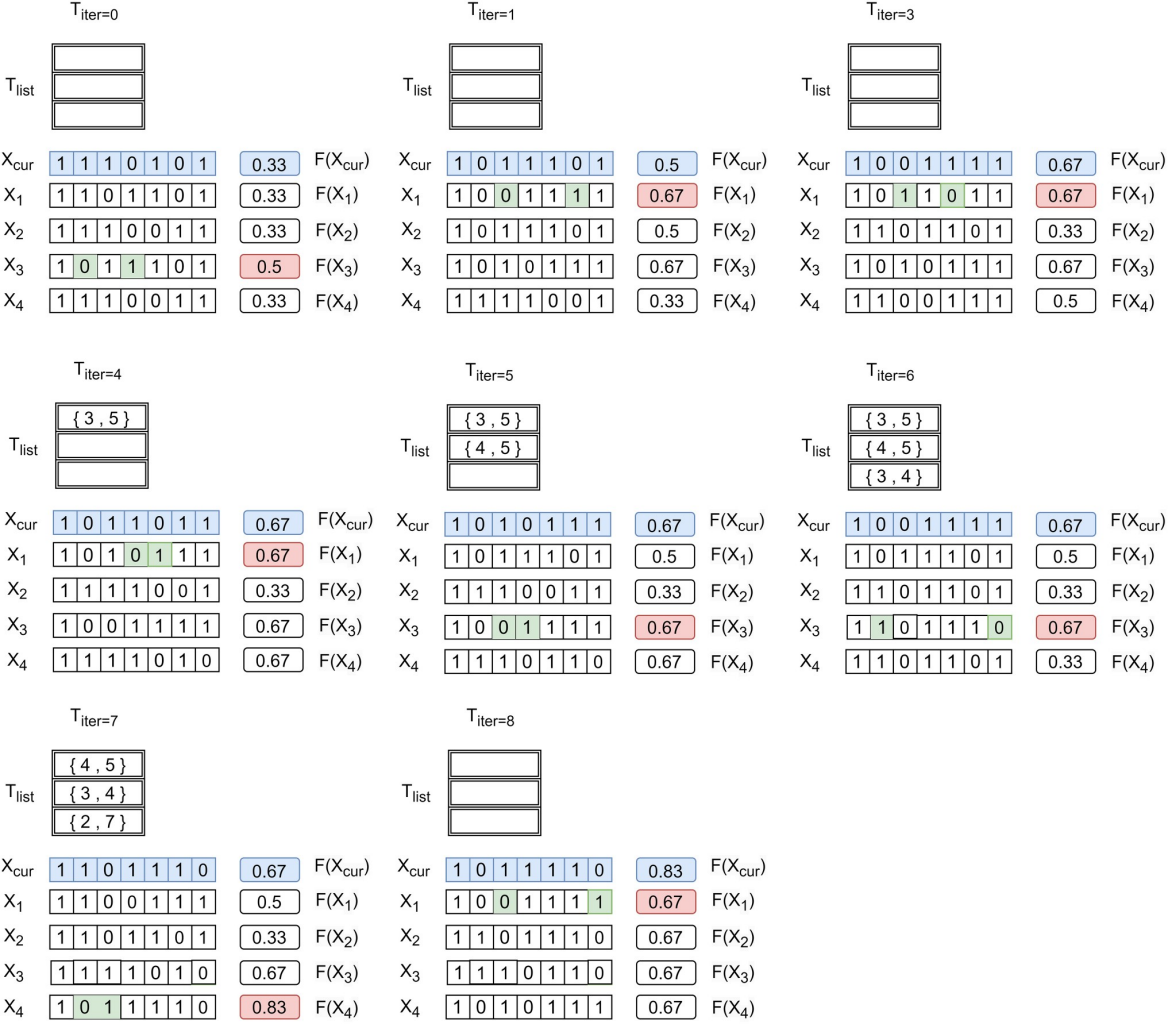
Illustration of the tabu search-based food web disintegration strategy. The blue row represents the current solution, *X*_1_, *X*_2_, *X*_3_, *X*_4_ represents the four different candidate solutions generated, and the right rectangle represents *F*(*X*_*ind*_). The red value is the optimal value of the objective function in one cycle, and its corresponding swap is noted as green.

Here we use a simple instance to illustrate the optimal disintegration strategy of food webs on the basis of tabu search. As shown in Fig 2, there are six species in this food web, denoted as nodes *v*_2_, …, *v*_7_, with the addition of virtual node *v*_1_. initialize *n* = 2, *L* = 3, *n*_*can*_ = 4, *T*_*list*_ = *NULL*, *T*_*iter*_ = 0, *T*_*max*_ = 8. Fig 3 shows the complete procedure for determining the optimal disintegration strategy. First, select two different nodes in *v*_2_, …, *v*_7_ randomly and assign their corresponding *x* value to 0 to generate the initial solution *X*_0_ = [1, 1, 1, 0, 1, 0, 1], i.e., delete node 4 and node 6, followed by making *X*_*cur*_, *X*_*opt*_ = *X*_0_. Then we generate four different candidate solutions by randomly swapping deleted and undeleted nodes, then calculate the degree of disintegration of the food web *F*(*X*_*ind*_). The blue rows in the figure represent the current solution, *X*_1_, *X*_2_, *X*_3_, *X*_4_ represent four different candidate solutions generated, and the right rectangle represents *F*(*X*_*ind*_). Find the *X*_*ind*_ corresponding to the largest *F*(*X*_*ind*_), note it as *X*_*k*_, and mark it in red. In the first iteration, *F*(*X*_3_) is maximum, so *X*_*k*_ = [1, 0, 1, 1, 1, 0, 1]. At the same time, mark the swap scheme green in this line, then *X*_*cur*_ = *X*_*k*_. Since *F*(*X*_*k*_) = 0.5 > 0.33, then *X*_*opt*_ = *X*_*k*_, *T*_*list*_ = *NULL*, *T*_*iter*_ = *T*_*iter*_ + 1. At this time, the optimal solution is [1, 0, 1, 1, 1, 1, 0, 1]. Then repeat the steps above. The list formed by the hollow rectangle in the figure represents the tabu list, which stores the swap sequences that are not available in the next generation of candidate solutions. The algorithm above continues until *T*_*iter*_ = 8 when the number of iterations reaches *T*_*max*_. At this point, we obtain the optimal solution [1, 0, 1, 1, 1, 1, 1, 0], which means deleting node 2 and node 7 maximizes the disintegration of the food web.

### Food web data

We analyzed quantitative food webs for 12 ecosystems of different sizes (Table 1, [30, 31]). The selection principles are as follows: (1) networks with species richness *S* > 20 were chosen to minimize mistakes owing to limited network size [32]; (2) each dataset was collected from different research to avoid the simultaneous occurrence of comparable networks from the same region (e.g., we randomly selected one network from the four sets of networks from Neuse Estuary in early summer 1997, late summer 1997, early summer 1998 and late summer 1998).

**Table 1:** Major characteristics of food webs used in the present research.

Food web	Original name	*S*	*C*_*n*_	*C*	*C*_*w*_	Reference
Crystal River	Crystal River (thermal)	21	61	0.138	0.074	[38]
Swartkops Estuary	Swartkops Estuary	25	104	0.166	0.090	[39]
Okefenokee Swamp	Okefenokee Swamp	26	132	0.195	0.077	[40]
Neuse Estuary	Neuse Estuary (early summer 1998)	30	77	0.086	0.057	[41]
Gulf of Maine	Gulf of Maine	31	332	0.345	0.148	[42]
Middle Atlantic Bight	Middle Atlantic Bight	32	375	0.366	0.156	[42]
Chesapeake Bay	Chesapeake Bay	36	122	0.094	0.068	[43]
Graminoids	Graminoids (wet)	66	793	0.182	0.033	[44]
Cypress	Cypress (dry)	68	554	0.120	0.058	[45]
Lake Oneida	Lake Oneida (pre-ZM)	74	1220	0.223	0.073	[46]
Mangroves	Mangroves (dry)	94	1339	0.152	0.036	[47]
Florida Bay	Florida Bay (dry)	125	1969	0.126	0.032	[48]

*S*, *C*_*n*_, *C*, and *C*_*w*_ represent the number of species, number of links, connectance and weighted directed connectance, respectively. Dry and Wet refer to food webs for dry and wet seasons, accordingly.

Food web data are available in the “enaR” package of R [30], S1 Data. Nodes represent the species, trophic group, functional group or system, and non-living components with energy stored. Edges represent the energy flux. Data for each food web include carbon biomass per taxonomic unit (*gCm*^−2^), carbon per unit time for imports, exports and respiration per taxonomic unit (*gCm*^−2^*day*^−1^) and carbon fluxes between pairs of taxonomic units (*gCm*^−2^*day*^−1^). The food webs we selected exhibited a wide range of network complexity, as measured by species richness (*S* = 21 − 125), number of connections (*C*_*n*_ = 61 − 1969), binary directed connectance (*C* = 0.079 − 0.354) and weighted directed connectance (*C*_*w*_ = 0.032 − 0.162). The binary directed connectance is a qualitative descriptor based on a binary network, *C* = *C*_*n*_/*S*^2^, which estimates the proportion of possible connections achieved between nodes. The weighted directed connectance indicates number of “valid” links in a quantitative food web *C*_*w*_ [33].

In addition, all substances in the food web come from primary producers who obtain substances from the external environment and transfer them to all other species through the food web [34, 35]. Therefore, we attach a virtual node to the network that points to all primary producers. Moreover, each species has an intrinsic energy loss that can be illustrated by adding a connection from each node to the virtual node. (Fig 2)

### Simulations of extinction

The topological approach based on secondary extinction is used in the present study, with the extinction threshold incorporated [12, 36]. The topological approach focuses only on the presence or absence of predator-prey relationships and considers only the qualitative food web structure. In this way, secondary extinction happens when a consumer loses all its energy sources [37]. The advantage of this method is that only the food web structure is required as input, simplifying its application in large and complicated food webs. However, it has limitations since it assumes that all species have the same baseline risk of extinction and that species will only disappear secondarily when all prey have been removed. More realistically, a species becomes extinct when energy flux input decreases below a critical level. The extinction threshold (*t*, [12]) is defined as the minimum energy required for the species to survive. After each removal, the percentage of extant energy to the original inflow energy, *P*(*i*), is recalculated for each species. If this fraction is equal to or less than the threshold (*P*(*i*) ≤ *t*), species *i* extincts secondarily. For example, *t* = 0.5 means that a species becomes extinct if the energy flux input of the species after removal is equal to or less than 50% of the initial intake, i.e., *P*(*i*) ≤ 0.5. In the conventional topological method, *t* is implicitly assumed to be equal to zero, and a species becomes extinct when its energy inflow is zero [1, 12, 13, 18, 49–51]. The extinction threshold can be seen as a way to adjust the vulnerability of consumers to the loss of resources.

To test our algorithm, we conducted simulations in which we removed one species in each step and recorded the proportion of secondary extinctions. Secondary extinctions can be direct (i.e., consumers go extinct after the removal of species with which they are directly associated) or indirect (i.e., consumers go extinct after species with which they are not directly associated are removed). This process is repeated until the extinction of all species. We compared several simple algorithms: (1) removing species in decreasing order of out-degree (OD), an approach that assumes that the species with the highest outward energy output is more important; (2) removing species with the greatest in-degree (ID) at each step, identifying keystone species in the opposite way to OD; (3) removing species in decreasing order of the sum of in-degree and out-degree (SD); (4) removing species in decreasing order of the product of in-degree and out-degree(PD); (5) algorithm based on weighted network eigenvectors (EIG, [18]); and finally, (6) removing according to the tabu search-based algorithm (TS) outlined above.

### Measurements and statistics

First, we recorded the proportion of secondary extinctions after removing each species. The x-axis of the secondary extinction curve (Fig 1, [1]) represents the proportion of primary extinctions, while the y-axis is the proportion of cumulative secondary extinctions. If no secondary extinction occurs after any primary extinction, then the extinct fraction will always match the removal fraction (1:1). However, if the loss of a species results in a subsequent secondary extinction, then the extinct fraction will surpass the removal fraction, and the resulting curve will be above the *y* = *x* line. The next one is the secondary extinction area (SEA) (Fig 6, [12]), which represents the area below the secondary extinction curve but above the *y* = *x* line. SEA is calculated as:

$$\begin{array}{c} SEA=\frac{\sum_{p=1}^{N}N_{p}}{N^{2}}-0.5 \end{array}$$ (3)

where *N* is the number of species in the initial food web, *p* is a specific number of primary extinction, and *N*_*p*_ is the number of extinction species. It can be inferred that SEA equals 0.5 when all species are extinct after the initial extinction and 0 when there is no more secondary extinction [18]. Thus the greater the SEA, the less robust the food web under species removal and the more effective the removal method. The last one is robustness (*R*_50_, [1, 13, 50, 52]), which is the minimum proportion of primary extinct species necessary to cause 50% of species in the food web to become extinct. The lower the *R*_50_ value, the more secondary extinctions and the lower the stability. We varied the threshold *t* from 5% to 95% by 5% and recorded the robustness of each *t* for each removal approach. By comparing the *R*_50_ values for different removal approaches, we can see how much the various removal methods destroy robustness and thus judge the effectiveness of various algorithms.

To determine if there is a statistically significant difference between the *R*_50_ (or SEA) obtained by the six algorithms OD, ID, SD, PD, EIG, and TS, we performed a one-way ANOVA with a threshold of *t* = 0.5. The ANOVA factors were simulated algorithms, with the null hypothesis that there is no significant difference among the *R*_50_ (or SEA) obtained by various algorithms. Accordingly, the alternative hypothesis is that there are significant differences. Then, we used Dunnett post hoc test, with the TS algorithm as the control group and other algorithms as the experimental group. It tested five null hypotheses that the *R*_50_ (or SEA) obtained by the TS algorithm did not vary significantly from the OD (or ID, SD, PD, EIG) algorithm. Rejecting it implies that removing species on the basis of tabu search leads to significantly different secondary extinction than other algorithms. That is, strategy based on tabu search is a keystone species identification tool that is either effective or ineffective (depending on whether it causes more or less secondary extinctions than other algorithms).

## Results

### Secondary extinct curve

**Fig 4:**
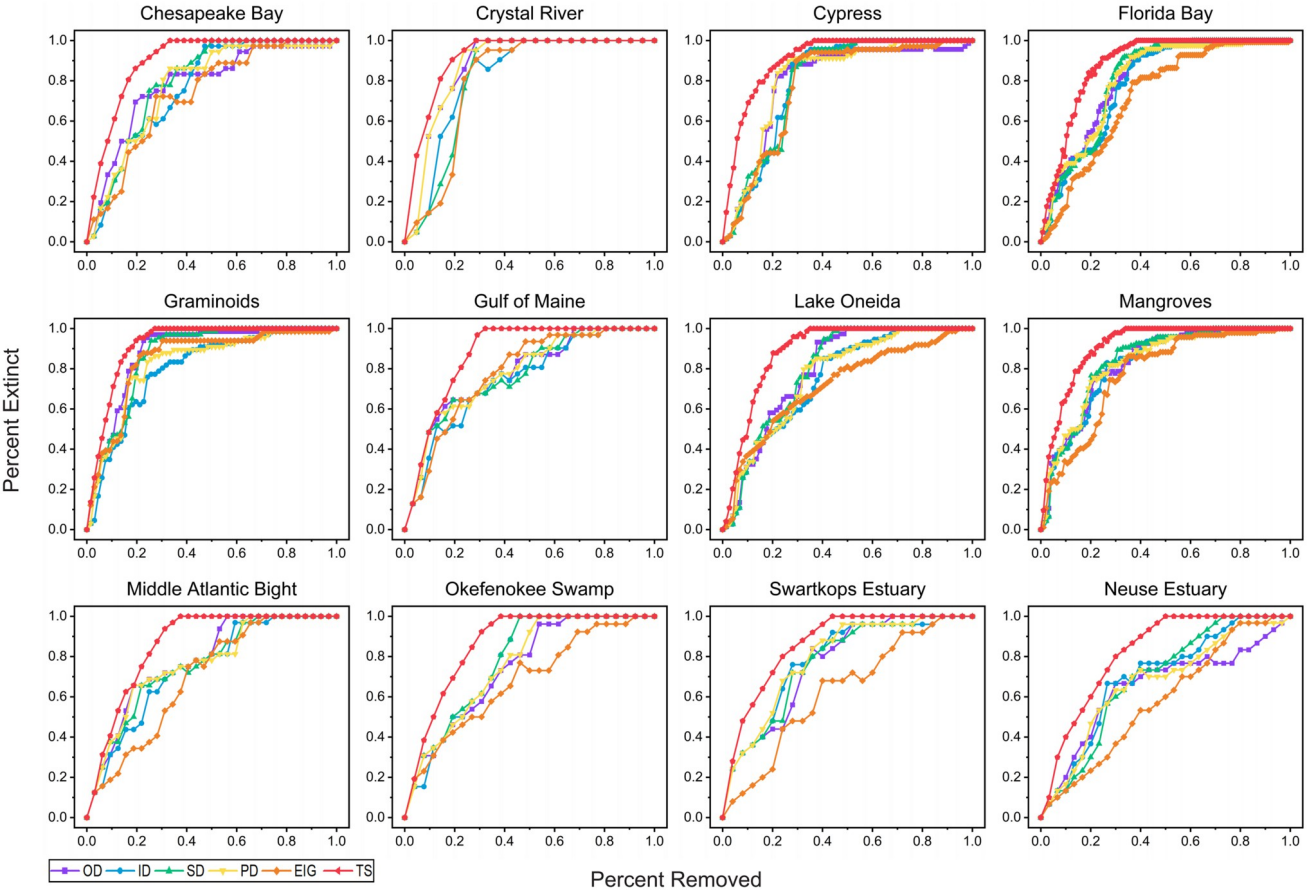
Relationship between primary and cumulative extinction in 12 food webs in order of species richness. ID, OD, SD, PD, EIG and TS represent the keystone species identification strategies based on in-degree, out-degree, sum of in-degree and out-degree, product of in-degree and out-degree, eigenvector, and tabu search, respectively. All algorithms were tested 10 times on each food web, and averages were taken as results.

The experimental results on the variation of the cumulative secondary extinction proportion with the primary extinction proportion are shown in Fig 4. A clear trend emerges in all 12 food webs secondary extinction curves: the secondary extinction curves obtained by the tabu search-based algorithm had a higher slope at the beginning, and the collapse of the corresponding food web occurred faster. In other words, the tabu search-based removal algorithm was more likely to result in more secondary extinctions than ID, OD, SD, PD, and EIG algorithms.

It can be concluded that if we remove those species identified by tabu search, the ecosystem will collapse faster than removal based on centrality or eigenvectors, which means that these species contributes more significantly to the ecosystem’s stability and can be considered keystone species. If a keystone species becomes extinct, more other species will go extinct secondarily [3–6]. In order to maintain the robustness of ecosystems, these species should be prioritized for protection.

### Secondary extinction area

**Fig 5:**
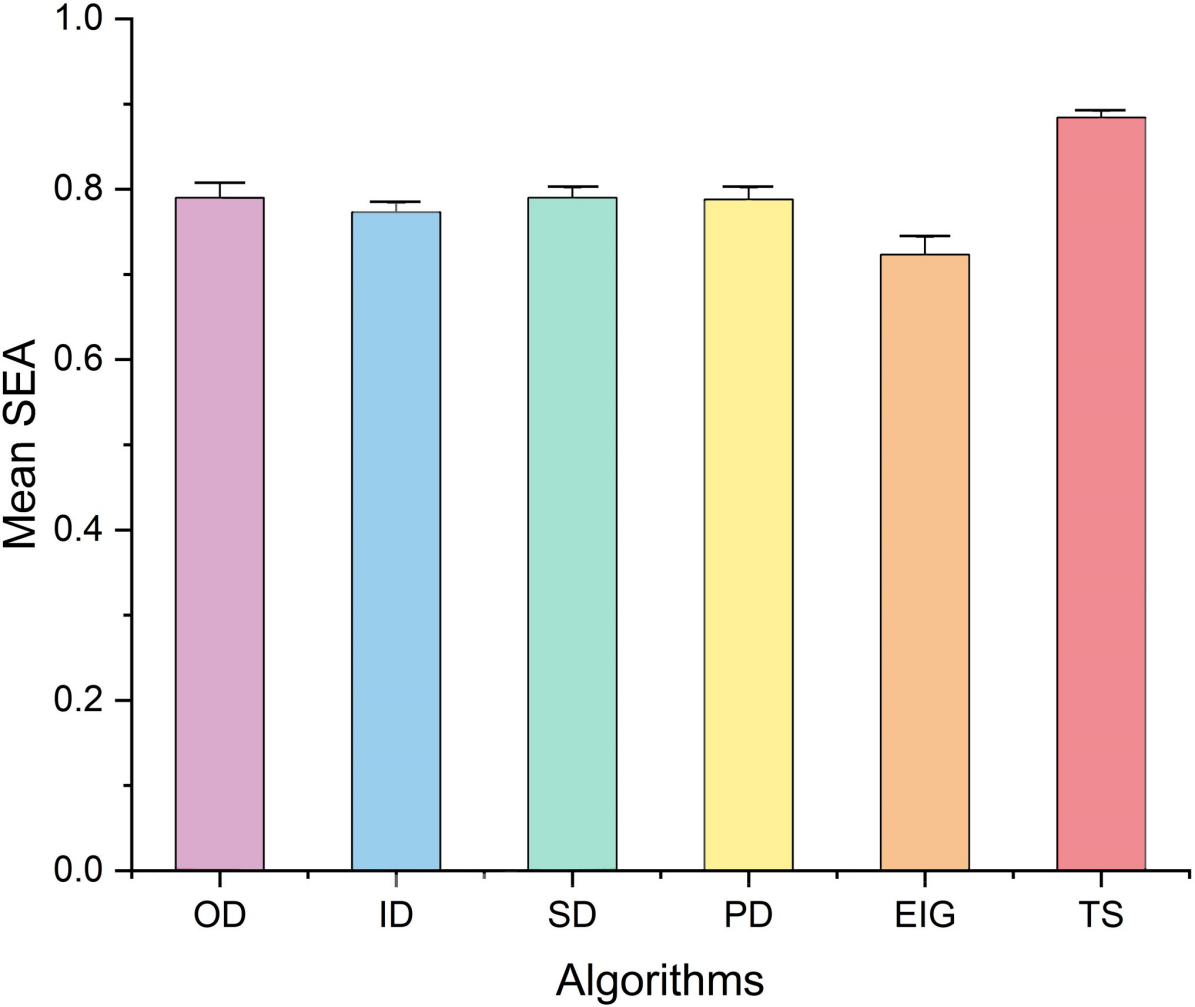
Mean SEA values(mean ± SEM) of each removal algorithm.

The SEAs obtained by various removal algorithms for the 12 food webs are shown in Table 2. In all cases, the species removal algorithm based on tabu search produced a higher SEA. To show the superior performance of the TS algorithm, we calculated the mean SEA values of various removal algorithms (Fig 5) with OD of 0.290±0.060, ID of 0.274±0.036, SD of 0.291±0.043, PD of 0.288±0.050, EIG of 0.223±0.073 and TS of 0.385±0.030. One-way ANOVA results demonstrated (F-value = 12.676, *p* < 0.001) that the simulations of the various algorithms produced significantly different SEAs. The fundings of Dunnett multiple comparisons (Table 3) further demonstrated that the TS algorithm caused significantly higher SEAs than the other algorithms. This implies that more secondary extinctions are owing to the removal of species according to the tabu search-based algorithm.

**Table 2:** Secondary extinction area(SEA, *t* = 0.5).

Food web	OD	ID	SD	PD	EIG	TS
Crystal River	0.374	0.322	0.315	0.376	0.302	0.415
Swartkops Estuary	0.267	0.283	0.272	0.291	0.130	0.363
Okefenokee Swamp	0.246	0.274	0.281	0.262	0.179	0.361
Neuse Estuary	0.149	0.200	0.190	0.171	0.082	0.320
Gulf of Maine	0.281	0.249	0.272	0.281	0.217	0.373
Middle Atlantic Bight	0.283	0.243	0.260	0.267	0.193	0.359
Chesapeake Bay	0.279	0.265	0.300	0.272	0.233	0.398
Graminoids	0.378	0.301	0.364	0.327	0.348	0.420
Cypress	0.297	0.304	0.301	0.315	0.281	0.410
Lake Oneida	0.294	0.248	0.294	0.263	0.217	0.393
Mangroves	0.326	0.317	0.336	0.327	0.274	0.415
Florida Bay	0.307	0.287	0.309	0.306	0.223	0.391

All algorithms were tested 10 times on each food web and averages were taken as results.

**Table 3:** Dunnett post hoc test results of secondary extinction area.

Methods				Confidence Interval	
Method1	Method2	Mean Difference	Significance	Lower	Upper
OD	TS	- 0.095	<0.01	- 0.148	- 0.041
ID	TS	- 0.111	<0.01	- 0.164	- 0.057
SD	TS	- 0.094	<0.01	- 0.148	- 0.041
PD	TS	- 0.097	<0.01	- 0.150	- 0.043
EIG	TS	- 0.162	<0.01	- 0.215	- 0.108

### Index of robustness

**Fig 6:**
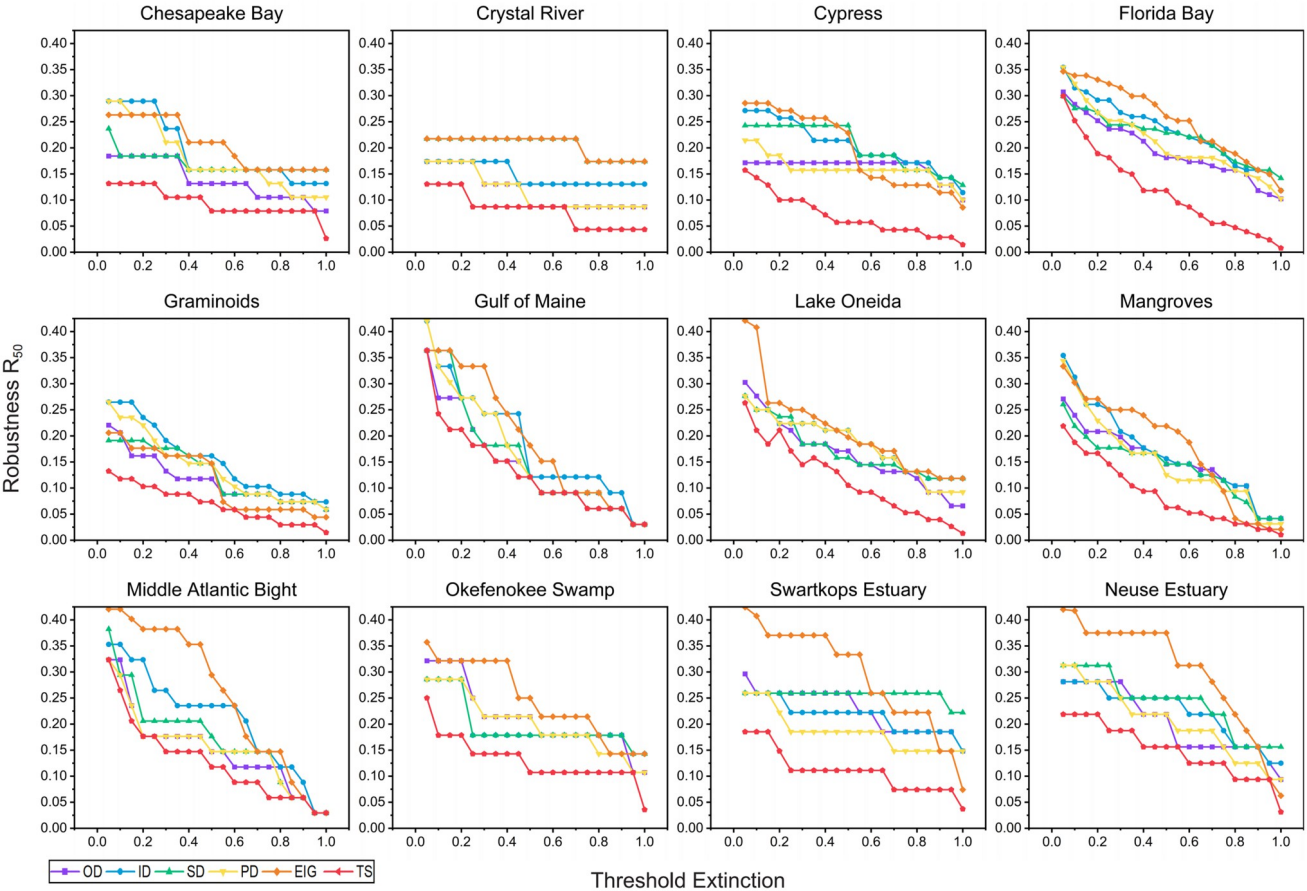
Relationship between extinction threshold (*t*) and Robustness (*R*_50_) in 12 food webs in order of species richness. The threshold *t* varied from 5% to 95% by 5%, and the robustness of each *t* is recorded for each removal approach. All algorithms were tested 10 times on each food web and averages were taken as results.

With the increase of extinction threshold, the *R*_50_ decreases for all removal conditions. As consumer sensitivity to energy loss increases, the removal of a smaller number of species can trigger an extinction of 50% species in the food web. The variation of *R*_50_ with energy threshold *t* for each food web is reported in Fig 6. A clear trend is also obvious in *R*_50_ curves of all 12 food webs: the keystone species identification algorithm based on tabu search tends to cause lower food web robustness. Except for the Gulf of Maine and Middle Atlantic Bight food webs, where it was less pronounced, overall, the food web robustness of species removal using the TS algorithm was lower than the other algorithms, regardless of the threshold *t*.

**Fig 7:**
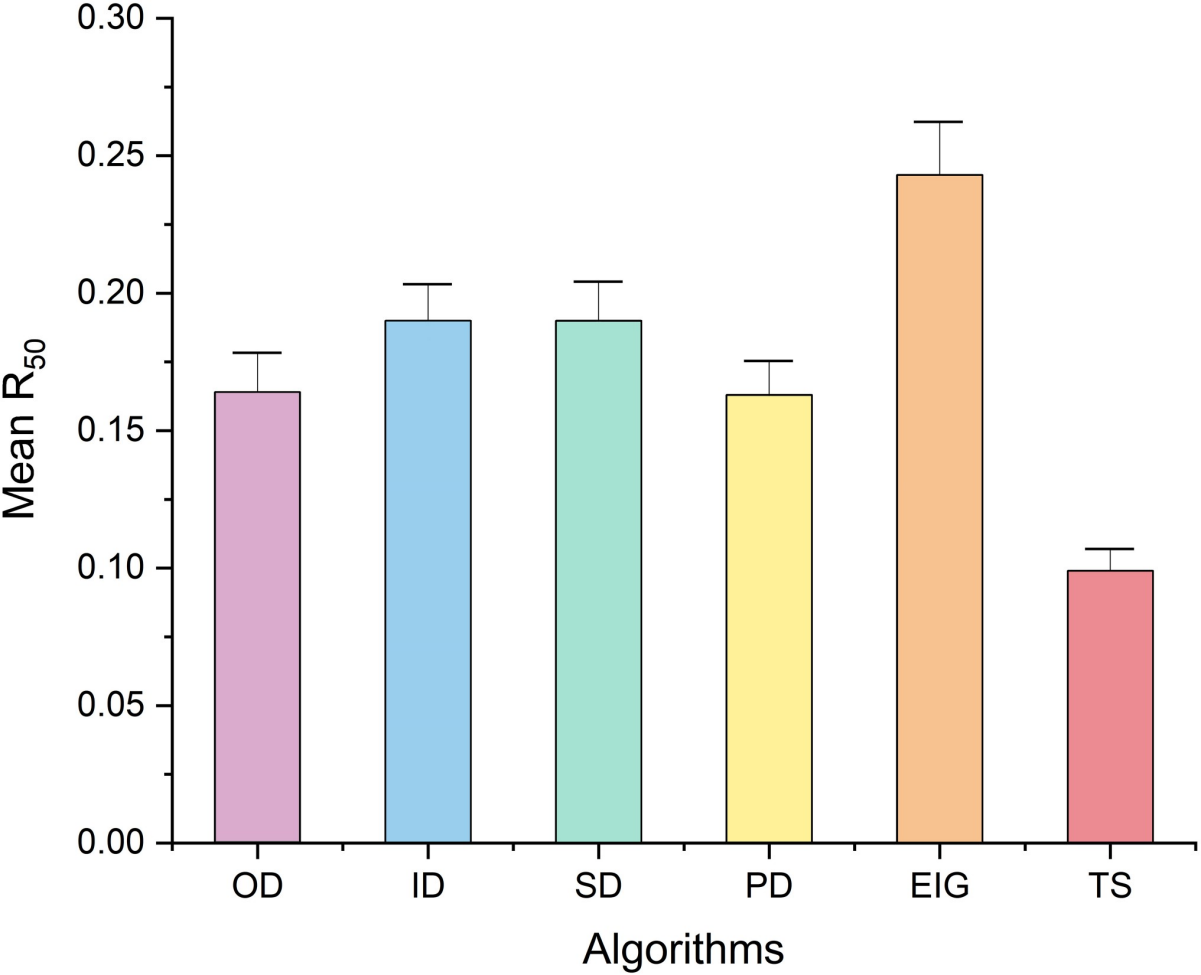
Mean *R*_50_ values(mean ± SEM) of each removal algorithm(*t* = 0.5).

In addition, we obtained the mean *R*_50_ values of various removal algorithms with *t* = 0.5 (Fig 7) to investigate the effect of different removal algorithms on *R*_50_ with OD of 0.164 ± 0.049, ID of 0.190 ± 0.044, SD of 0.190 ± 0.047, PD of 0.163 ± 0.041, EIG of 0.243 ± 0.065, and TS of 0.100 ± 0.029. One-way ANOVA revealed that the species removal algorithms had a substantial effect on the robustness *R*_50_: the six removal algorithms produced quite different *R*_50_ values in the topological simulations (F-value = 11.922, *p* < 0.001). In addition, the TS algorithm had the lowest mean *R*_50_ value, which was considerably different from the results of other algorithms (Dunnett post hoc test, Table 4). To summarize, these findings demonstrate that the tabu search-based algorithm always performs better than the centrality and eigenvector-based algorithms in identifying keystone species, which play an essential role in maintaining ecosystem stability.

**Table 4:** Dunnett post hoc test results of robustness.

Methods				Confidence Interval	
Method1	Method2	Mean Difference	Significance	Lower	Upper
OD	TS	0.064	0.06	0.015	0.114
ID	TS	0.090	<0.01	0.040	0.139
SD	TS	0.091	<0.01	0.041	0.140
PD	TS	0.064	0.07	0.014	0.113
EIG	TS	0.143	<0.01	0.094	0.193

## Discussion

We developed an optimization model for the disintegration strategy in quantitative food webs and obtained the optimal strategy using tabu search. Through comparing with other traditional centrality-based and eigenvector-based methods, we obtained larger SEA values as well as lower *R*_50_ values, and verified the effect of the strategy on food web disintegration. It is demonstrated that the method based on tabu search can identify the most destructive near-optimal species in the food web, i.e., the keystone species in the ecosystem by global search.

The difference between the tabu search-based and other algorithms also caught our attention. Contrary to the concept of centrality that has been emphasized so far, the findings obtained by various algorithms emphasize that the most damaging species identified by a global search are more effective than the individual species’ importance ranking approach. Classical centrality measures such as in-degree and out-degree can identify important nodes in binary food webs [12], but cannot effectively identify the truly critical nodes in quantitative food webs. In particular, although it has been shown to perform similarly to genetic algorithms in binary food webs [18], the eigenvector-based algorithm does not perform well in quantitative food webs.

Srinivasan et al. [53] have shown that many realistic removal sequences are unlikely to result in large-scale species loss and that the extinction of vulnerable species does not necessarily result in the extinction of other species. Therefore, it is difficult to determine the effect of removing species that have a minor direct impact on the structure of the food web. Tabu search-based algorithms can incorporate these species into a “global search” of the food web, using “memory” to guide the local search beyond the local optimum when confronted with a local optimum. Even though the removal of such species may temporarily lead to a more stable food web structure, the tabu search will remove it to achieve the final global optimum.

Previous studies have used centrality as a criterion for identifying keystone species and have suggested that species of high centrality are more influential than others and play an important role in structural maintenance. Nevertheless, it is difficult to determine how many species ought to be defined as keystone species in a given ecosystem. Previous search has not given specific numbers or percentages of species that ought to be defined as keystone species [3, 4, 10], only priority lists based on centrality [54], with ranking indicating the significance of species in maintaining biodiversity. However, the high centrality of species A, B, and C, respectively, does not mean that their combination is important for the ecosystem. In comparison, the strategy based on tabu search can discover which species groups are essential to the ecosystem, which may help to determine which species should be defined as keystone species.

It should be noted that, in contrast to centrality-based approaches, our approach requires complete information about the food web structure. However, in many realistic situations, it is hard to gather such precise data. Therefore, our approach is expected to be extended to situations where information is incomplete or inaccurate. Furthermore, our study is based on a topological approach where a species becomes extinct when the inflow energy decreases below the extinction threshold and assumes that consumers cannot switch from one prey species to another. However, it is known this type of adaptive behavior is available in the ecosystem, and several studies have estimated the robustness of food webs in dynamic models [16, 54–57]. Therefore, the next challenge is to identify keystone species in dynamic food web models with the help of tabu search.

## Conclusion

The Earth’s ecosystem plays an essential role in maintaining ecological balance and protecting the regional environment [58], but it is experiencing a severe decline in biodiversity. The decrease in biodiversity will cause dramatic changes in ecosystem processes and functions. Therefore, it is of great importance to conserve biodiversity, especially to protect keystone species [59]. In the present study, we developed an optimal model of disintegration techniques in quantitative food webs. To solve the model and obtain the optimal disintegration strategies, an innovative strategy based on tabu search was proposed. Then, we estimated the secondary extinction of each food web by topological simulation and evaluated the secondary extinction effect using the secondary extinction curve, SEA, and *R*_50_. The results suggest that our method is effective compared with conventional methods. Future research is suggested to concentrate on (1) expanding our method to food webs with incomplete or inaccurate information, and (2) identifying keystone species in dynamic food web models using tabu search.

## Supporting information

S1 Data (JSON)
